# Alleviation of Cadmium Stress in Rice Seedlings Inoculated with *Enterobacter tabaci* 4M9 (CCB-MBL 5004)

**DOI:** 10.21315/tlsr2024.35.1.6

**Published:** 2024-03-30

**Authors:** Saidu Abdullahi, Hazzeman Haris, Kamarul Zaman Zarkasi, Amir Hamzah Ghazali

**Affiliations:** 1 School of Biological Sciences, Universiti Sains Malaysia, 11800 USM Pulau Pinang, Malaysia; 2 Department of Botany, Ahmadu Bello University, 810001 Zaria, Nigeria

**Keywords:** Cadmium Tolerant Bacteria, Catalase, Electrolyte Leakage, Plant Growth Enhancement, Superoxide Dismutase, Bakteria Tahan Kadmium, Katalase, Kebocoran Elektrolit, Pertumbuhan Anak Benih Padi, Superoksida Dismutase

## Abstract

The growth of crop plants is greatly affected by the increased toxicity of metals. Luckily, certain beneficial bacteria can potentially reduce the effects of metal stress and promote the growth of the host plants. Many species of bacteria were reported as heavy metal tolerant and plant growth promoting, with very little or no report available concerning *Enterobacter tabaci* as heavy metal tolerant plant growth promoting. The present study aimed to evaluate the potential of Cadmium (Cd) tolerant *Enterobacter tabaci* 4M9 (CCB-MBL 5004) to alleviate heavy metals stress and enhance the growth of rice seedlings grown under Cd stress conditions. Rice seedlings were grown in Yoshida medium supplemented with different concentrations of Cd and inoculated with 4M9. The results showed that the inoculum tested successfully reduced oxidative stress in the seedlings by reducing the electrolyte leakage (EL) and increasing catalase (CAT) and superoxide dismutase (SOD) activities in the inoculated seedlings compared to the control counterparts. The results also revealed a significant increase in plant growth, biomass, and chlorophyll content of inoculated rice seedlings compared to the control. In general, the Cd tolerant *E. tabaci* 4M9 confers heavy metal alleviation and thereby improves the growth and survival of rice seedlings under Cd stress conditions. Therefore, the findings stated the potential of 4M9 for alleviating heavy metal stress and promoting the development of inoculated rice seedlings if accidentally grown under Cd-contaminated conditions.

## Abstract (ms)

Pertumbuhan tanaman sangat dipengaruhi oleh peningkatan ketoksikan logam. Sungguhpun begitu, terdapat bakteria bermanfaat tertentu yang berpotensi untuk mengurangkan kesan tekanan logam tersebut dan menggalakkan pertumbuhan tanaman perumah. Terdapat banyak spesies bakteria telah dilaporkan sebagai rintang logam berat dan berupaya menggalakkan pertumbuhan tanaman perumah, sungguhpun begitu terdapat sangat sedikit atau tiada laporan tersedia mengenai *Enterobacter tabaci sebagai* bakteria penggalak pertumbuhan dan pada masa sama rintang logam berat. Oleh itu, kajian ini dijalankan bertujuan untuk menilai potensi bakteria tahan terhadap kadmium (Cd) *E. tabaci* 4M9 (CCB-MBL 5004) untuk mengurangkan tekanan logam berat dan meningkatkan pertumbuhan anak benih padi yang ditanam di bawah keadaan tekanan Cd. Anak benih padi ditanam dalam medium Yoshida ditambah dengan kepekatan Cd yang berbeza dan diinokulat dengan 4M9. Keputusan menunjukkan bahawa inokulum yang diuji berjaya mengurangkan tekanan oksidatif anak benih melalui pengurangan kebocoran elektrolit (EL) dan meningkatkan aktiviti katalase (CAT) dan superoksida dismutase (SOD) dalam anak benih padi yang diinokulasi berbanding dengan rawatan kawalan. Hasil kajian menunjukkan bahawa terdapat peningkatan ketara dalam pertumbuhan tumbuhan, biojisim, dan kandungan klorofil anak benih padi yang diinokulasi berbanding kawalan. Secara amnya, *E. tabaci* 4M9 yang tahan Cd berjaya pengurangan tekanan logam berat dan seterusnya dapat meningkatkan pertumbuhan dan kemandirian anak benih padi di bawah keadaan tekanan Cd. Oleh itu, penemuan ini telah berjaya menunjukkan potensi 4M9 untuk mengurangkan tekanan logam berat dan menggalakkan perkembangan anak benih padi berinokulasi jika ditanam secara tidak sengaja di bawah keadaan tercemar Cd.

**Highlights**

- *Enterobacter tabaci* 4M9 (CCB-MBL 5004) is a potential cadmium (Cd) tolerant isolate which able to alleviate heavy metals stress (Cd) and enhance the growth of rice seedlings.
- *E. tabaci* 4M9 (CCB-MBL 5004) successfully reduced oxidative stress in the host plants via reducing the electrolyte leakage (EL), increasing catalase (CAT) and superoxide dismutase (SOD) activities in the inoculated rice seedlings.
- The isolate could potentially alleviate heavy metal stress and promote the development of rice seedlings if accidentally grown under Cd-contaminated conditions.

## INTRODUCTION

The amount of potentially toxic substances released into the environment increases with the alarming growth in the global population (Jabeen *et al*. 2022). Heavy metals, unlike organic contaminants, are not degraded or destroyed, and their total concentrations persist for an extended period (Kour *et al*. 2021). According to Etesami (2018), heavy metals in soils have detrimental effects on the growth of staple crops and can pose a severe health risk to humans. There was a report by Marquez *et al*. (2018) that heavy metals, such as cadmium (Cd), copper (Cu) and lead (Pb), have been identified to have adverse effects on the growth of rice (*Oryza sativa* L.). Among the heavy metals that are reported in the list of top 20 hazardous substances, Cd is among the most toxic metals to humans (Tchounwou *et al*. 2012). Cd induces nutritional deficiency in plants, and exposure to higher levels can reduce the rate of photosynthesis, nutrient and water uptake, chlorosis, growth inhibition and death (Khalid *et al*. 2017; Ma *et al*. 2016). It is reported by Ismael *et al*. (2019) that Cd is the only metal with health risk effects to both humans and animals at plant tissue concentrations that are non-phytotoxic. This shows that plants growing at sites with low Cd contamination may not show any toxicity symptoms but accumulate Cd higher than allowed levels for humans.

The vulnerability of many plants to several adverse environmental situations such as metal toxicity and other deleterious conditions leads to oxidative damage and stress, which may on the other hand affect many of their biological processes through the generation of reactive oxygen species (ROS) (Tchounwou *et al*. 2012; Ojuederie & Babalola 2017; Xie *et al*. 2019). The ROS may be generated by the plant’s normal metabolic activities such as respiration and photosynthesis, however, in stress conditions, there is a metabolic imbalance that can cause the formation of excess ROS, causing oxidative damage and stress (Rossatto *et al*. 2017). The ROS which includes hydroxyl radicals (OH^−^), superoxide (O_2_^−^), hydrogen peroxide (H_2_O_2_), and singlet oxygen (O_2_), can severely damage cell structure, organelles, and affect several other important activities in plants (Kim *et al*. 2017). Excessive ROS can also cause lipid peroxidation and electrolyte leakage (EL) that leads to permanent damage to membranes as well as membrane integrity and functions (Khan 2015a). It was observed by Zhao *et al*. (2021) that cadmium stress caused damage to the structure of cell membrane due to the effect on peroxidation of inner membrane of sassafras seedlings. Hassan *et al*. (2020) reported a significant reduction in the growth of two sorghum cultivars and increased EL, H_2_O_2_ concentration and malondialdehyde (MDA) content in both cultivars due to Cd stress.

Alleviation and management of the damage are usually achieved by developing an antioxidant system to maintain homeostasis through the production of enzymatic antioxidants including catalase (CAT), superoxide dismutase (SOD), ascorbate peroxidase, peroxidase (POD) etc. and non-enzymatic antioxidants like carotenoids, tocopherols, glutathione, ascorbate, etc. (Khan 2015a; Xie *et al*. 2019). SOD is the first to act in the face of defence regarding the elimination of ROS, causing the dismutation of the superoxide radicals into H_2_O_2_ (Rossatto *et al*. 2017). CAT and the ascorbate peroxidase (APX) take part in the conversion of H_2_O_2_ into oxygen and water (Kim *et al*. 2017). SOD, CAT and POD are the primary antioxidant enzymes, and when plants are under heavy metal stress, their activity increases gradually with increasing metal concentration (Zhao *et al*. 2021). The enzyme system may be destroyed when the heavy metal concentration becomes too high leading to a decrease in activity.

Plants develop several defence mechanisms for tolerance of heavy metals toxicity, but at certain points and limits, the mechanisms may fail, and the survival of the plants is seriously affected (Tiwari & Lata 2018). Plant-microbe relationships may play a significant role in enhancing the plant’s ability to produce antioxidants and other defensive mechanisms against excessive ROS and oxidative stress (Islam *et al*. 2016). Different studies have shown that selected plant growth promoting rhizobacteria (PGPR) were able to reduce different oxidative stresses in plants and increase antioxidant activities (Islam *et al*. 2016; Khan 2015a; Swapnil *et al*. 2016). The beneficial microbes could be considered as one of the most promising and eco-friendly alternatives for safe and sustainable crop-management practices against heavy metal exposure issues (Tiwari & Lata 2018). Some recent studies revealed that inoculation of plants with specific PGPR could alleviate metal-induced toxicity and enhance the production of plant biomass grown in metal-contaminated soils (Farh *et al*. 2017; Mallick *et al*. 2018; Rizvi & Khan 2017). Bacteria with such beneficial characteristics could be used as a source of biofertiliser for crops under stress conditions. Many species of bacteria were reported as heavy metal tolerant and plant growth promoting, with very little or no report available concerning *E. tabaci* as heavy metal tolerant plant growth promoting. Thus, the present study was conducted aiming to evaluate the influence of locally isolated Cd tolerance bacteria *E. tabaci* 4M9 (CCB-MBL 5004), towards alleviation and protection of inoculated rice seedlings against Cd stress conditions. Additionally, growth enhancement of *E. tabaci* 4M9 inoculated rice seedlings under Cd stress conditions was observed.

## MATERIALS AND METHODS

### Plant Material and Bacterial Strains

Rice (*Oryza sativa* L.) seeds, MR220 variety, provided by the Malaysian Agricultural Research and Development Institute (MARDI), were used in this study. The seeds were treated with cadmium-tolerant *Enterobacter tabaci* 4M9 (CCB-MBL 5004) previously isolated from the rhizosphere soil of *Mimosa pudica* growing naturally on ex-tin mining soil (Abdullahi *et al*. 2021), which possessed plant growth-promoting traits including phosphate and potassium solubilisation, nitrogen fixation and production of indole acetic acid (IAA), ammonia, siderophore and 1-aminocyclopropane-1-carboxylic acid (ACC) deaminase.

### Preparation of Bacterial Inoculants, Rice Seeds and Cd Solutions

The bacterial strain was cultured in 100 mL nutrient broth in 250 mL Erlenmeyer flasks for 24 h on a shaking incubator (Protech Orbital Shaker, model 720 D, Techlab Scientific) at 180 rpm and 30°C. The cells were harvested by centrifugation at 8,000 rpm for 15 min at 4°C and washed twice with sterile distilled water. The cell pellets were then resuspended in sterile distilled water and adjusted to an absorbance of 1 at 600 nm (Khan 2015a) using a spectrophotometer (T60 visible spectrophotometer, PG instruments). The rice seeds were surface sterilised by immersing in 70% ethanol for 30 min, washed three times with sterile distilled water, soaked in 2% sodium hypochlorite solution for 10 min, and finally rinsed thoroughly with sterile distilled water (Ndeddy Aka & Babalola 2016). Sterilised seeds were soaked in the bacterial solutions (for bacterial inoculation treatments) or sterile distilled water (for control treatments) for 4 h before placing them in sterile Petri dishes with two moistened filter papers at the bottom. The seeds were left to germinate in the plates under dark conditions at room temperature for 4 to 7 days. The concentrations of Cd tested were in the range of 0 mg/L, 5 mg/L and 10 mg/L. The concentrations of Cd tested were based on a preliminary result that rice seedlings could sustain growth up to 10 mg/L of Cd (unpublished data).

A total of six different treatment combinations involving Cd and *E. tabaci* 4M9 were carried out in a completely randomised design with 10 replications each. Uniformly germinated seeds from plates were transferred to test tubes (20 cm in height and 2.5 cm in diameter) containing 10 mL sterilised Yoshida medium (Yoshida *et al*. 1976). Sterilised and cooled Yoshida medium of about 50°C to 60°C was supplemented with appropriate concentrations of filter-sterilised Cd solutions and poured into the tubes. The bacterial suspension or sterile distilled water was aseptically added near the base of the plants on day three after transplanting. Seedlings were left to grow in respective test tubes under controlled conditions for 15 days.

### Determination of Electrolyte Leakage

The oxidative damage due to Cd stress on rice seedlings was measured in EL according to Ahmad *et al*. (2014) and Gontia-mishra *et al*. (2016). A total of 100 mg of leaves of rice seedlings from each inoculation treatment were cut into small pieces and transferred into test tubes containing 10 mL distilled water and shaken on an incubator shaker for 4 h at 150 rpm. The electrical conductivity (EC) of the solution was measured using the Gro Line Soil EC and temperature tester before (C1) and after autoclaving at 121°C for 20 min (C2). The EL of the samples was determined using the following formula:


$$\%\;\text{EL}=\frac{\text{C}1}{\text{C}2}\times 100$$

where, C1 = EC of the sample before autoclaving; and C2 = EC of the sample after autoclaving.

### Determination of Antioxidant Enzymes Activities

A total of 200 mg fresh leaf of treated rice seedlings was homogenised in 2 mL of 0.1 M potassium phosphate buffer (pH 7.8) with 0.1 mM EDTA to obtain crude extract. The mixture was centrifuged at 15,000 ×*g* for 20 min at 4°C. The supernatant was stored in aliquots at −20°C for CAT and SOD antioxidant enzyme activity assay. For CAT, the activity was calculated and expressed in millimoles of H_2_O_2_ per minute per gram fresh weight using the extinction coefficient of H_2_O_2_ (40 mM^−1^ cm^−1^ at 240 nm) (Mosa *et al*. 2017). At the same time, the activity of SOD was determined by using the nitro blue tetrazolium chloride (NBT) method, according to Mosa *et al*. (2017).

### Determination of Growth, Biomass and Root Colonisation

Treated rice seedlings were monitored for growth responses, which include root length (cm), shoot length (cm), root dry weights (g), and shoot dry weights (g). Chlorophyll estimation of the leaf was done using the dimethyl sulfoxide DMSO method (Hiscox & Israelstam 1978). A total of 0.1 g of fresh leaves were cut into pieces, placed in tubes, and 10 mL dimethyl sulfoxide was added. The tubes were kept under dark conditions for 72 h at 4°C, and the absorbance was measured spectrophotometrically at 645 nm and 663 nm using dimethyl sulfoxide as blank before calculating the chlorophyll contents. The standard plate count technique determined the colony-forming units (CFU) of viable microbial cells colonising the treated roots. Samples were rinsed serially in sterile saline water to detach the microbes inhabiting the root surfaces. A total of 100 μL of dilutions were spread plated on a nutrient agar medium and incubated for 24 h to 48 h. Using dilution and mass correction factors, plates containing 30 to 300 colonies were selected, counted, and calculated for CFU.

### Statistical Analysis

Data were subjected to analysis of variance (ANOVA) to test for significant differences among treatments. Where a significant difference exists, Tukey’s HSD was used to separate and compare means at a 5% probability level. Statistical analysis was carried out with IBM SPSS Statistics version 26.

## RESULTS

### Oxidative Stress and Antioxidant Enzyme Activities of Inoculated Rice Seedlings

**Figure 1:**
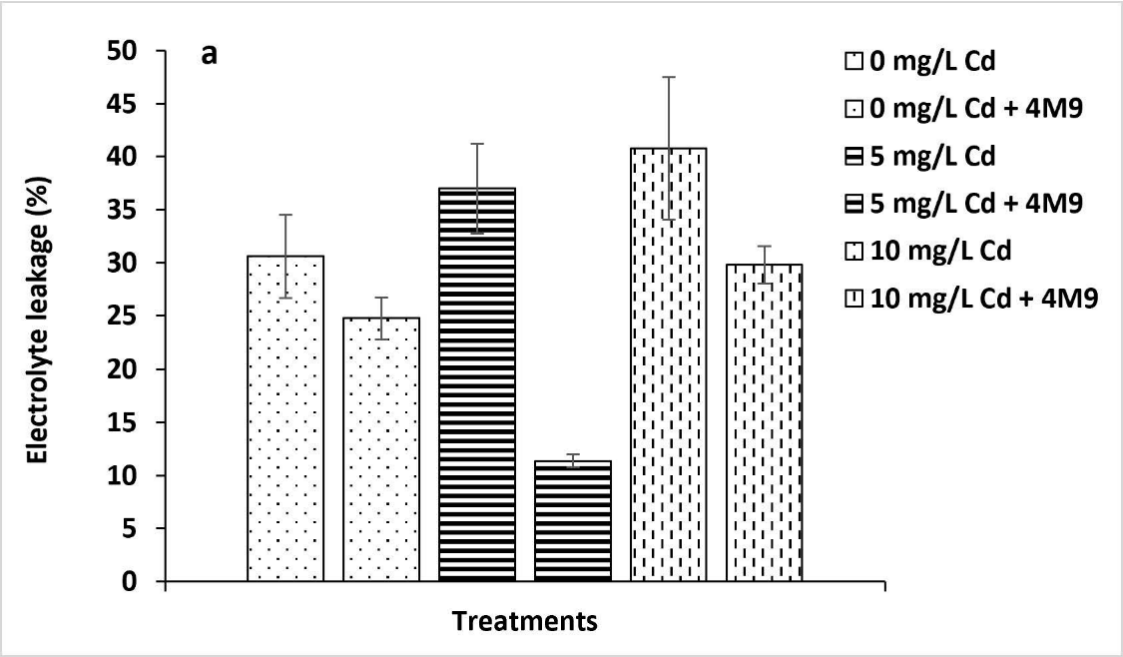

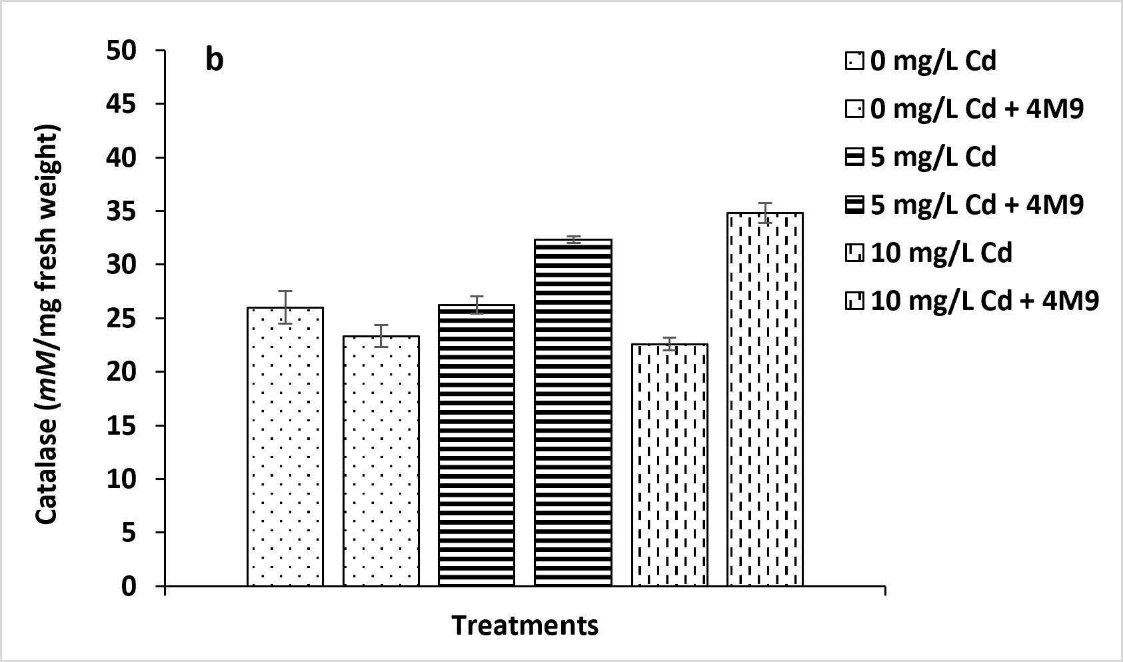

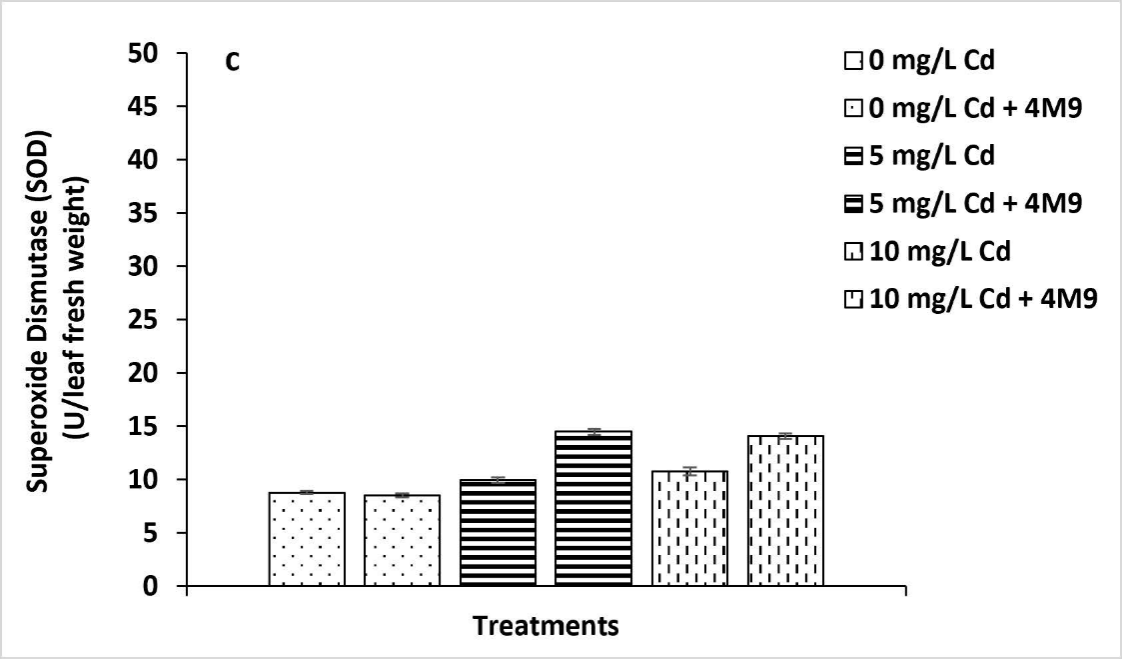
Effect of *E. tabaci* 4M9 on (a) electrolyte leakage; (b) catalase and (c) superoxidase dismutase activities of rice seedlings under Cd stress conditions after 15 days of growth.

The influence of bacterial inoculation on oxidative stress and the degree of damage on rice seedlings caused by Cd stress in the form of EL is presented in Fig. 1a. The treatments were shown to cause oxidative stress in the leaves of the rice plants, and the oxidative stress increased in response to increasing concentrations of Cd added. There was a significant decrease in the EL values in 4M9 inoculated plants, which is a clear indication of the stability of the membrane. The % EL in the plants inoculated with 4M9 (Fig. 1a) decreased by 27% under the highest concentrations of Cd. Uninoculated rice seedlings showed up to 41% of EL for 10 mg/L Cd. The results proved the evidence of Cd stress alleviation due to inoculation of *E. tabaci* 4M9. The CAT activity determined in the leaves of rice seedlings exposed to Cd was significantly higher in plants inoculated with 4M9 compared to the uninoculated control (Fig. 1b). A similar scenario was observed in the case of SOD activity under Cd (Fig. 1c) stress conditions. Strains 4M9 increased CAT activity by 54% under the highest Cd stress, respectively, and increased SOD activity by 31%. It was also observed that the inoculated controls without the addition of Cd had minor activities in all cases. An increase in CAT and SOD activities indicated a strong response of the seedlings inoculated with 4M9 towards coping with oxidative stress generated by exposure to Cd.

### Growth and Biomass of Inoculated Rice Seedlings

**Figure 2:**
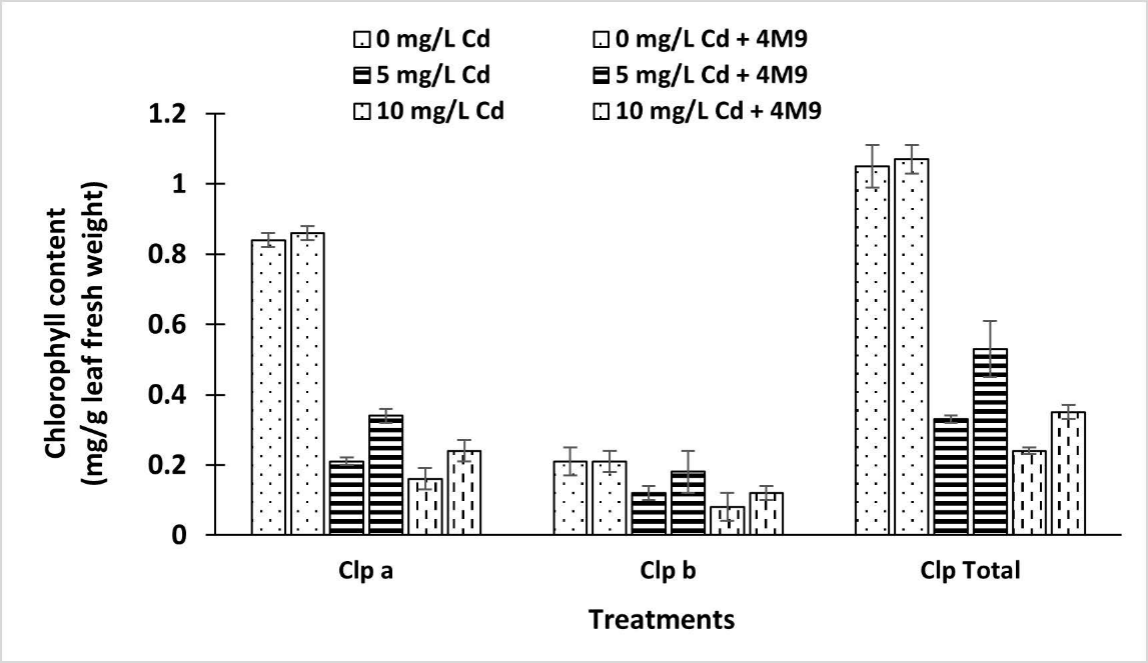
Chlorophyll content in rice seedlings inoculated with *E. tabaci* 4M9 under Cd stress conditions after 15 days of growth.

The growth of rice seedlings inoculated with 4M9 under Cd (0 mg/L, 5 mg/L and 10 mg/L) stress was observed and presented in Table 1. For both inoculated and uninoculated seedlings, all growth parameters were noted to decline as affected by increasing concentrations of Cd compared to control treatments (0 mg/L Cd). However, rice seedlings’ parameters with bacterial inoculation treatments (7.7 × 10^6^ – 1.5 × 10^7^ CFU/g) showed an increasing trend compared to uninoculated treatments under similar concentrations of Cd tested. Additionally, the control treatments inoculated with 4M9 at 0 mg/L Cd successfully promoted better plant growth than the rest of the treatments and proved the plant growth potentials of the isolates. Results in Table 1 also showed that bacterial strain 4M9 could tolerate and alleviate Cd stress effects on rice seedlings and promote plant growth compared to the control. Inoculating 4M9 to rice seedlings treated with up to 10 mg/L Cd promoted increased root length from 6.07 cm to 7.73 cm (27%) and root dry weight from 2.60 to 3.70 mg (42%). The bacterial strains significantly increased chlorophyll a, chlorophyll b and total chlorophyll contents of inoculated rice seedlings despite the high stress of Cd (Fig. 2). Entire chlorophyll content was significantly increased by 46% for 4M9 compared to uninoculated rice seedlings. Response of 4M9 towards alleviating Cd effects was more promising at 5 mg/L Cd.

## DISCUSSION

Plants are constantly being exposed to several stress factors in the environment, including heavy metals contamination of soils which affects plant growth and development (Thomas & Archana 2023). The adverse environmental stress factors generally induce the accumulation of ROS, which can cause severe oxidative damage, including EL to plants. Cadmium negatively affects the growth of plants, and it enters plants so easily because of its high solubilisation in water, thereby affecting seed germination, transpiration and photosynthesis, and other metabolic activities (Zafar-ul-Hye *et al*. 2020). Plants have defense mechanisms against oxidative damage, which are activated during stress to regulate the toxic levels of ROS (Caverzan *et al*. 2016; Azeem *et al*. 2022). Apart from that, the interaction between the plant and certain beneficial bacteria can also alleviate the stress factor caused by heavy metal exposure (e.g., Cd) and enhance the growth of the host plant (Ajmal *et al*. 2021; Thomas & Archana 2023). In this study, the EL from the plants is one factor studied that progressively increased with increasing concentrations of Cd in the rhizosphere zone of the host plants. Higher EL values were recorded for the Cd treated rice seedlings compared to the controls. However, the 4M9 inoculated rice seedlings had successfully lowered the EL percentage which proved Cd stress alleviation on the host plants due to the bacterial inoculation treatments. Similar reports on reducing EL in microbes-treated plants under various metals stress conditions were also reported earlier by Das and Sarkar (2018) and Khan (2015a).

In this study, the CAT and SOD antioxidants of leaves varied between 4M9 inoculated and uninoculated rice seedlings grown in the presence of Cd. Both CAT and SOD values were higher in inoculated treatments than in uninoculated controls, which indicated potential of 4M9 to remediate heavy metals’ stress effects on the host plants. Earlier findings by Islam *et al*. (2016) also showed that the antioxidant defense system of lentil plants treated with copper-resistant bacteria improved by producing significantly higher antioxidant enzymes, including ascorbate peroxidase, superoxide dismutase, catalase and guaiacol peroxidase. The result is also in agreement with several others (Azeem *et al*. 2022; Singh *et al*. 2022). Jabeen *et al*. (2022) inoculated hydroponically grown rice plants with *B. cereus* and observed improved plant growth (increased plant biomass, chlorophyll contents, relative water content), increased enzymatic activities (catalase, superoxide dismutase, peroxidase, etc.) and reduced MDA content in both roots and leaves of rice plants under Cd stress. Their results suggested that the *B. cereus* can be used as a biofertiliser for rice cultivation in Cd-polluted soils.

In this study, the rice seedlings treated with Cd recorded reduced plant biomass. However, different conditions were recorded for rice seedlings inoculated with Cd-tolerant plant growth-promoting bacteria *E. tabaci* 4M9. Treated seedlings recorded significantly increased growth and plant biomass despite being under Cd stress. Earlier findings by Das and Sarkar (2018) and Farh *et al*. (2017) stated a reduction in plant growth due to exposure to heavy metals. However, the development was improved for plants inoculated with heavy metal tolerant beneficial bacteria. Rizvi and Khan (2017) also stated their findings on the biotoxic impact of heavy metals on wheat and highlighted phytotoxic effects of Cd, Cr and Cu on wheat increased with increasing rates of the metal tested. However, in contrast, the wheat inoculated with *Pseudomonas aeruginosa* CPSB1 had better growth and yields compared to control under similar heavy metals stress conditions. The bacterial strain (4M9) persisted in the rhizosphere and rhizoplane of the rice seedlings, which indicated their colonisation ability while carrying out heavy metals stress alleviation functions. The beneficial effects of any heavy metal resistant plant growth-promoting bacteria can include reducing heavy metals toxicity and stress effects on host plants (Yin *et al*. 2019). The inoculation treatment accelerated root development, which may, in turn, result in better access to nutrients and faster initial growth, leading to enhanced remediation and sustainable plant biomass production (Khan *et al*. 2015b; Kour *et al*. 2021).

The result presented also showed that leaf chlorophyll contents of the treated plants decreased significantly due to Cd stress. Toxicity of heavy metals (e.g., Cd) can affect photosynthesis by causing distortion in the ultrastructure of the chloroplast, preventing the synthesis of photosynthetic pigment in chlorophyll content and enzymes of the Calvin cycle (Dharni *et al*. 2014). Zhao *et al*. (2021) and Jabeen *et al*. (2022) reported that the physiological effects of Cd on the growth and biomass of plants are mainly due to its high mobility in water and toxicity to photosynthetic organs. However, our findings have shown that inoculation of the bacterial strain effectively ameliorates the metals’ adverse effects on chlorophyll contents. An increase in the chlorophyll contents of the host plants due to inoculation of 4M9 was observed for both Cd treated and control conditions. Our findings had successfully proven that 4M9 inoculation had successfully improved not only plant resistance towards Cd stress conditions but also promoted the chlorophyll content of the host plants. Locally isolated 4M9 can protect the host plant from Cd toxicity effects through several mechanisms, including reducing EL and increasing antioxidant enzyme production. Plant growth attributes enhanced due to 4M9 inoculation include shoot and root length, chlorophyll contents, and dry biomass. The results presented were in accordance with earlier results presented by Abdullahi *et al*. (2021), that 4M9 has various plant growth-promoting traits, including phosphate and potassium solubilisation, nitrogen fixation, and production of indole acetic acid (IAA), ammonia, siderophore and 1-aminocyclopropane-1-carboxylic acid (ACC) deaminase.

## CONCLUSIONS

In general, bacterial inoculant *E. tabaci* 4M9 (Cd tolerant) confers heavy metal stress resistance and improved growth and survival of rice plants under Cd stress. The inoculum tested successfully reduced oxidative stress in the rice plants due to Cd contaminations. This was evident by their influence in reducing EL, increasing antioxidant enzymes (CAT and SOD) activities, and enhancing the growth of 4M9 inoculated rice seedlings under Cd stressed conditions. The combined properties of *E. tabaci* 4M9 in terms of heavy metal tolerance and plant growth-promoting traits can be exploited further and developed as a component of biofertiliser in metal-contaminated soils especially in rice fields for effective growth enhancement and metal stress alleviation.

## Figures and Tables

**Table 1:** Growth characteristics and root colonisation of rice seedlings inoculated with *E. tabaci* 4M9 under Cd stress conditions after 15 days of growth.

Cd (mg/L)	Inoculation (4M9)	Root length (cm)	Shoot length (cm)	Root dry weight (mg)	Shoot dry weight (mg)	CFU/g
0	−	4.86 ± 0.19^de^	28.67 ± 0.44^a^	3.57 ± 0.23^b^	16.10 ± 0.49^b^	–
	+	6.67 ± 0.60^bc^	29.50 ± 0.76^a^	4.97 ± 0.39^a^	19.73 ± 2.54^a^	7.8 × 10^6^
5	−	4.50 ± 0.50^e^	10.10 ± 0.31^c^	2.67 ± 0.08^c^	6.37 ± 0.23^d^	–
	+	8.33 ± 0.44^a^	13.76 ± 0.43^b^	4.03 ± 0.26^b^	11.63 ± 0.19^c^	7.7 × 10^6^
10	−	6.07 ± 0.52^cd^	8.17 ± 0.17^c^	2.60 ± 0.35^c^	7.80 ± 0.65^d^	–
	+	7.73 ± 0.50^ab^	8.83 ± 0.17^c^	3.70 ± 0.25^b^	9.93 ± 0.64^cd^	1.5 × 10^7^

*Notes:* Values are presented as means ± standard error. Values with different superscripts along the columns are significantly different (*p* < 0.05). –: uninoculated; +: inoculated.
